# Microbial Enrichments Contribute to Characterization Of Desert Tortoise Gut Microbiota

**DOI:** 10.1007/s00248-025-02557-6

**Published:** 2025-06-17

**Authors:** Elaina M. Blair, Noa J. Margalith, Michelle A. O’Malley

**Affiliations:** 1 Department of Chemical Engineering, University of California, Santa Barbara, CA 93106 USA; 2 Department of Chemical & Biomolecular Engineering, University of California, Berkeley, CA 94720 USA; 3 Department of Bioengineering, University of California, Santa Barbara, CA 93106 USA; 4 Joint BioEnergy Institute (JBEI), Emeryville, CA 94608 USA

**Keywords:** Microbial consortia, Lignocellulose, Anaerobic, Enzyme, Tortoise

## Abstract

Desert tortoises play ecologically significant roles, including plant seed dispersal and mineral cycling, and yet little is known about microbial members that are critical to their gut and overall health. Tortoises consume recalcitrant plant material, which their gut microbiota degrades and converts into usable metabolites and nutrients for the tortoise. Findings from tortoise gut microbiomes may translate well into biotechnological applications as these microbes have evolved to efficiently degrade recalcitrant substrates and generate useful products. In this study, we cultivated microbial communities from desert tortoise fecal samples following a targeted anaerobic enrichment for microbes involved in deconstruction and utilization of plant biomass. We employed 16S rRNA amplicon sequencing to compare cultivated communities to initial fecal source material and found high abundances of Firmicutes and Bacteroidota typically associated with biomass deconstruction in all cultivated samples. Significantly decreased microbial diversity was observed in the cultivated microbial communities, yet several key taxa thrived in lignocellulose enrichments, including *Lachnospiraceae* and *Enterococcus*. Additionally, cultivated communities produced short-chain fatty acids under anaerobic conditions, and their growth and metabolic output provide evidence of their viability in the initial fecal communities. Overall, this study adds to the limited understanding of reptilian herbivore microbiota, and offers a path towards biotechnological translation based on the ability of the cultivated communities to convert lignocellulose directly to acetate, propionate, and butyrate.

**Supplementary Information:**

The online version contains supplementary material available at 10.1007/s00248-025-02557-6.

## Introduction

Tortoise populations significantly contribute to their habitat, as they are involved in mineral cycling, building burrows to shelter themselves and other animals, seed dispersal, and additional key aspects of maintaining and restoring ecosystems [1]. Better understanding these reptiles can guide in strategies that promote their protection, especially those at risk for population decline [2]. It may also provide insight into what enables these tortoises to accomplish their environmental roles, including lignocellulose breakdown. From a biotechnological standpoint, this is useful in identifying potential avenues and microbial members that can be used for more efficient biomass degradation and conversion to products of interest. Most tortoises subsist mainly on a diet of plants, including grasses, flowers, fruit, and legumes [3, 4], but general knowledge regarding the tortoise gut microbiome is limited, which is not surprising given the scarcity of microbial data sourced from reptilian herbivores in general [5]. Data on their microbial gut communities are especially lacking, which hinders insight into disease etiologies and resilience, especially those related to climate change. For example, within the Joint Genome Institute’s Integrated Microbial Genomes & Microbiomes database [6], there are no metagenomes for host-associated Reptilia. While several reptilian-associated microbial isolate genomes are available, including for tortoises [7, 8], these provide limited insight into the wild-type diversity, unique taxa, and metabolic underpinnings that happen inside the guts of these reptiles.

Amplicon sequencing has been performed for a few different tortoise species [5, 9, 10], yet studies on desert tortoises have focused more on nasal communities than on the gut [11–13]. Nasal communities have provided valuable information on microbial colonization associated with upper respiratory tract disease since this disease has significantly hindered tortoise populations [14]. However, gut microbial communities play critical roles in the immune system and overall health, including via interactions with other parts of the body, such as the brain (e.g., via flow of microbial metabolites) [15], and thus, the gut microbiome should not be overlooked. Recent progress has been made in uncovering the presence of anaerobic gut fungi and their capacities in the tortoise gut [16, 17]. Anaerobic fungi are associated with biomass degradation [18], but the anaerobic fungi found in tortoise guts are less degradative than anaerobic fungi found in mammalian guts (e.g., there are substantially fewer carbohydrate-active enzymes encoded in tortoise-associated anaerobic gut fungal transcriptomes) [17]. Since tortoises consume plant biomass, bacteria and other microbial members within the tortoise gut must play a major role in biomass breakdown, and this microbiome may provide a source for novel biomass-degrading microbes and enzymes.

Past tortoise-associated microbial studies have mainly sequenced DNA directly from host-associated samples [9, 12, 19], meaning microbial members may be present but may or may not be metabolically active and proliferating. One study cultivated microbes from desert tortoise samples associated with the nose and hindgut, but the scope of this study was limited to microbes that thrived in the presence of oxygen (aerobes) [11],which necessarily selects against obligate anaerobes that would thrive in the gut [20]. Cultivating communities from environmental samples enables identification of viable microbes, though the members cultivated typically represent only a small portion of the starting viable community. This is largely due to challenges in identifying culture conditions, media, and appropriate environmental replication strategies that support growth of diverse microbiome members [21], yet high-throughput methods in cultivation (termed “culturomics”) are helping tackle this challenge [22].

However, high-throughput cultivation methods are not always necessary, particularly when the goal is to enrich for a particular type of community function. For example, since desert tortoises consume plant biomass [23], they host lignocellulose-degrading microbes within their gut – many or most of which are likely to be obligate anaerobes [24]. Additionally, lignocellulose-degrading communities can generate short-chain fatty acids, which are an essential nutritional source for animal hosts and help sustain a healthy gut [25], and also serve as value-added biological products on their own [26]. The purpose of this study was to better characterize the desert tortoise gut microbiome, with an emphasis on enriching and identifying members of the lignocellulose-degrading gut community. Hence, we cultivated microbial communities from captive tortoise feces on grass and identified viable microbial members that were enriched under the lignocellulosic culture conditions employed, many of which likely contributed to the conversion of lignocellulose to fatty acids.

## Methods

### Culture Media Used for Microbial Growth

Microbial communities were grown using M2 media [27], except the trace element solution was swapped (equal volume) with the trace mineral supplement from ATCC (MD-TMS, made in-house). Hemin and vitamin solutions were 0.2 µm filtered and added post-autoclaving. Hungate tubes containing 9 mL M2 media and 0.1 g reed canary grass as a substrate (1 mm milled) were autoclaved, and then 0.1 mL of 100 × hemin solution (final hemin concentration of 1 mg/L) and 0.1 mL of 100 × vitamin solution (ATCC MD-VS; final concentration of 0.1% v/v) were added.

### Microbial Community Dilution and Inoculation from Fecal Samples

Fecal samples from two desert tortoises (*Gopherus agassizii*; named Mojave and Pancake) were collected on May 19, 2023, from the Santa Barbara Zoo. Mojave was a male desert tortoise, with an age estimated to be between about 50 and 60 years; he had been living in captivity for at least 16 years at the time of sampling. Pancake was a female tortoise, 22 years old, who had been at the zoo for 4 years (she was with a private owner prior to that). These tortoises were fed a diet consisting of kale, collard greens, dandelion, cilantro, and a high-fiber commercial feed (Mazuri® Tortoise LS Diet). They also had regular access to browse, grass, and hay. The tortoise diet was supplemented with calcium (1/8 teaspoon of Repashy Supercal NoD, 3 times per week). For dilutions from Pancake’s feces, 10 mL of M2 culture media (no grass) were added to 1 g of feces while bubbling with supplemented CO_2_. This mixture was vortexed, and 1 mL was transferred via a sterile syringe and needle to a Hungate tube containing 9 mL M2 media with 0.1 g reed canary grass. This Hungate tube was inverted to mix and was used to inoculate four biological replicates; 1 mL was taken via syringe and needle and used to inoculate each new “Dilution 1” Hungate tube containing 9 mL M2 media with 0.1 g reed canary grass. One further dilution was performed by taking one of the Dilution 1 replicates and transferring 1 mL from it into new Hungate tubes containing 9 mL M2 media with 0.1 g reed canary grass; this was done for three biological replicates, termed “Dilution 2.” The Dilution 1 biological replicate used to inoculate Dilution 2 cultures was not incubated in the remainder of the experiment but was only used for inoculation purposes.

For dilutions from Mojave’s feces, since more starting fecal sample was available, 3 g of feces were used; 25 mL M2 media were added to the feces in a Falcon tube, while bubbling with CO_2_. This mix was vortexed, and 1 mL was transferred to a Hungate tube as before. This tube was used to inoculate Dilution 1 biological replicates, and Dilution 2 replicates were diluted from one of the Dilution 1 biological replicates, the same way as with the other tortoise fecal sample dilution and inoculation. The remaining fecal sample material from Pancake and Mojave was stored at –80 °C until DNA extraction.

### Microbial Community Cultivation and Incubation

To maximize identification of diverse microbes, we incubated communities at two different temperatures: 30 °C and 39 °C. The higher temperature (39 °C) was chosen because similar types of enrichment studies from mammalian herbivore gut or fecal samples have incubated microbial cultures at that temperature [28, 29], and the lower temperature (30 °C) was chosen since tortoises have lower body temperatures than mammalian herbivores. This temperature selection is supported by a recent study that isolated anaerobic gut microbes (specifically anaerobic gut fungi) from tortoises and found optimal incubation temperatures of 30 °C and 39 °C for different taxa [16]. Hungate tubes diluted from Mojave’s feces were incubated at 30 °C, while cultures diluted from Pancake’s feces were incubated at 39 °C (the set of enrichments (either from Mojave or Pancake) incubated at each temperature was chosen at random). After the initial culture growth, cultures were transferred to fresh media and incubated for 4–6 days for three passages (enrichments from Pancake’s feces were typically passaged every 4 days, and enrichments from Mojave’s feces were typically passaged every 5 days). Passaging frequency was chosen based on microbial growth (assessed via pressure measurements). For passaging, 1 mL of the growing culture was transferred via syringe and needle to a new Hungate tube containing 9 mL M2 media with 0.1 g reed canary grass.

### Culture Harvesting and DNA Extractions

At the end of the third passage, 1 mL of the culture was removed and frozen at –80 °C for supernatant sample analysis. The remaining culture was vacuum filtered through a 0.22-µm PVDF membrane (MilliPore Sigma, cat no. GVWP02500) to separate cells and grass from the culture media (many cells were likely attached to the grass particles). The filter, covered with grass and cells, was transferred to a DNeasy PowerSoil Pro kit lysis tube (Qiagen) and stored at –80 °C until DNA extraction. For extractions from fecal samples, approximately 0.1–0.2 g of fecal material was added to a DNeasy PowerSoil Pro kit lysis tube. For all samples, DNA extractions were performed using the DNeasy PowerSoil Pro kit, according to manufacturer protocol, except with a minor adjustment to the bead beating process. Specifically, 800 µL CD1 was added to each lysis tube; tubes were vortexed, and then samples were lysed via bead beating (Biospec Mini-BeadBeater-16), with 15 s on the bead beater, followed by 1 min on ice, repeated 3 times. For the last step, genomic DNA was eluted in 100 µL Solution C6. DNA concentrations were generally in the low hundreds of nanograms per microliter. DNA was then cleaned using the Zymo Research DNA Clean & Concentrator kit, following manufacturer protocol; cleaned DNA was eluted in 100 µL H_2_O.

### Library Preparation and 16S rRNA Gene Sequencing

Extracted DNA from eighteen microbial communities, with 3 biological replicates per condition (fecal samples, Dilution 1 enrichments, and Dilution 2 enrichments), was prepared for sequencing using the Illumina Nextera CD Index Kit. Standard Illumina primers were used to amplify the V3–V4 region of the 16S rRNA gene via PCR (forward primer: 5’ TCGTCGGCAGCGTCAGATGTGTATAAGAGACAGCCTACGGGNGGCWGCAG; reverse primer: 5’ GTCTCGTGGGCTCGGAGATGTGTATAAGAGACAGGACTACHVGGGTATCTAATCC). The samples were sequenced using the Illumina MiSeq v3, 600 cycle kit (paired end, 300 bp forward reads and 300 bp reverse reads), with PhiX added (~ 10% aligned). Approximately 1 million reads (including both forward and reverse reads) were sequenced per sample.

### Sequencing Analysis

Sequencing data was analyzed using QIIME 2 v. 2023.5 [30]. Reads were trimmed using DADA2 [31], where reverse reads were truncated at 219 bp because quality scores dropped off after that point; forward reads were truncated at 300 bp. Reads were classified into amplicon sequence variants (ASVs), and ASVs with < 10 reads summed across all samples were removed. Mitochondrial and chloroplast sequences were also removed based on taxonomic classification. Statistics on read counts before and after filtering are presented in Supplemental Tables 1–2; analyses were performed on filtered reads shown in the final column of Supplemental Table 1 (after DADA2 pipeline, removal of low abundance ASVs, and removal of chloroplast and mitochondrial sequences).

The filtered table had a total of 4645 ASVs; feature read counts totaled at least 20,000 per sample, and thus, no samples were removed due to low counts. An average of ~ 800 ASVs was obtained for fecal samples from each tortoise, and the average was between 200 and 400 ASVs for each of the dilutions (~ 400 for Mojave Dilution 1, ~ 300 for Mojave Dilution 2, and ~ 200 for both sets of Pancake enrichments). Alpha and beta diversity metrics were calculated at the level of ASVs, with samples rarified to 20,000 reads. These metrics were calculated using the QIIME 2 [30] “core-metrics-phylogenetic” command with the filtered table described above and the rooted tree (obtained from the QIIME 2 command “align-to-tree-mafft-fasttree” [32, 33], which used filtered representative ASV sequences from the samples). Faith’s phylogenetic diversity [34] was used to assess alpha diversity, with the Kruskal–Wallis [35] test used to calculate pairwise *p*-values and *q*-values. Beta diversity was assessed via Bray–Curtis and Jaccard distances [36].

Taxonomy was classified based on training [37, 38] with the Illumina primers employed using the Silva database (Silva 138 SSURef NR99 full-length sequences) [39, 40], with minimum length set to 100 and maximum length set to 600. ANCOM-BC [41] was used to identify taxa that were significantly enriched in the cultivated communities; the filtered table with 4645 ASVs was separated into two tables based on the individual tortoise sampled from, and taxonomy was assigned as described above. Each table was analyzed with ANCOM-BC; significance was set to *p* < 0.001, but results were filtered to include only those with *q* < 10^−5^; analyses were performed at the species level. ANCOM-BC results were calculated using QIIME 2 [30] with the commands “composition ancombc” [41] and “composition da-barplot.” Taxa with an average of fewer than 200 feature counts in the enrichment samples (for that tortoise) were filtered out.

### Metabolic Output via High-Performance Liquid Chromatography (HPLC)

Supernatant samples from each microbial enrichment culture were prepared for HPLC analysis by adding 30 µL of 50 mM H_2_SO_4_ to 270 µL supernatant sample; acidified samples were vortexed and incubated at room temperature for approximately 10 min. Samples were then centrifuged at 21,000 g for 5 min at 25 °C; any pellet was avoided, and samples were filtered through a 0.22 µm filter into a polymer insert for HPLC analysis. Standards for acetate, propionate, and butyrate were prepared in water at concentrations between 0.1 and 2 g/L. These samples were acidified and centrifuged similar to the other samples, but they were not filtered prior to transferring into the polymer insert for HPLC analysis. Samples and standards were run on the Agilent 1260 Infinity, with autosampler unit, using the Bio-Rad Aminex HPX-87H column. Mobile phase flowrate (5 mM H_2_SO_4_) was set to 0.6 mL/min, and column temperature was set to 60 °C. Acetate and propionate concentrations were measured using a refractive index detector, while butyrate was measured with a variable wavelength detector.

## Results

**Fig. 1:**
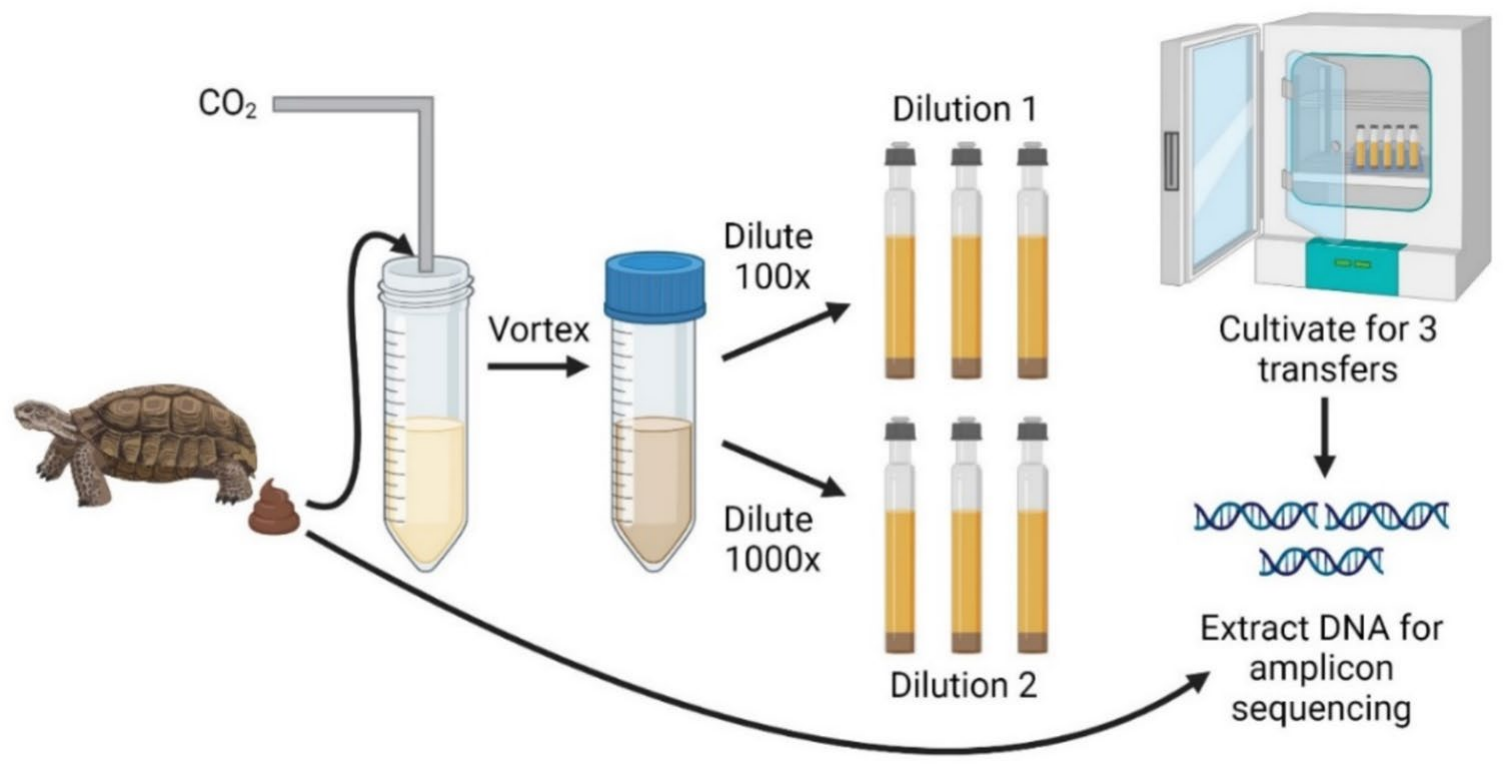
Microbial communities are cultivated from tortoise fecal samples. Communities were cultivated for three passages prior to harvesting for DNA extractions and 16S rRNA gene sequencing. This figure was created with BioRender.com

**Fig. 2:**
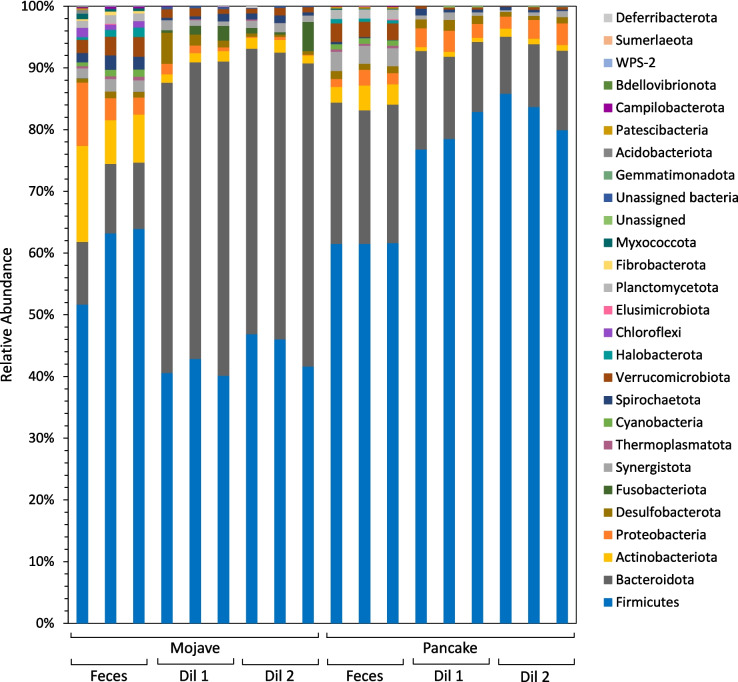
Phylum level taxonomy shows more diversity in desert tortoise fecal samples than cultivated communities and high abundance of Firmicutes and Bacteroidota in all samples. “Dil 1” and “Dil 2” refer to the microbial enrichment starting dilutions (Dilution 1 and Dilution 2). Mojave and Pancake refer to the names of the individual desert tortoises from which fecal samples were taken

Microbial communities were enriched from tortoise feces on lignocellulosic biomass for multiple transfers and analyzed via 16S rRNA gene sequencing (Fig. 1). We utilized two different starting dilutions from the fecal samples and compared the membership of these communities to the starting fecal samples via sequencing of the V3–V4 region of the 16S rRNA gene. Clear differences were found between cultivated and uncultivated microbial communities, and high abundances of Firmicutes and Bacteroidota were seen in all samples (Fig. 2), which aligns well with other studies from tortoise microbiomes [5, 42].

### Tortoise Feces Contain Greater Diversity than Microbial Enrichments

**Fig. 3:**
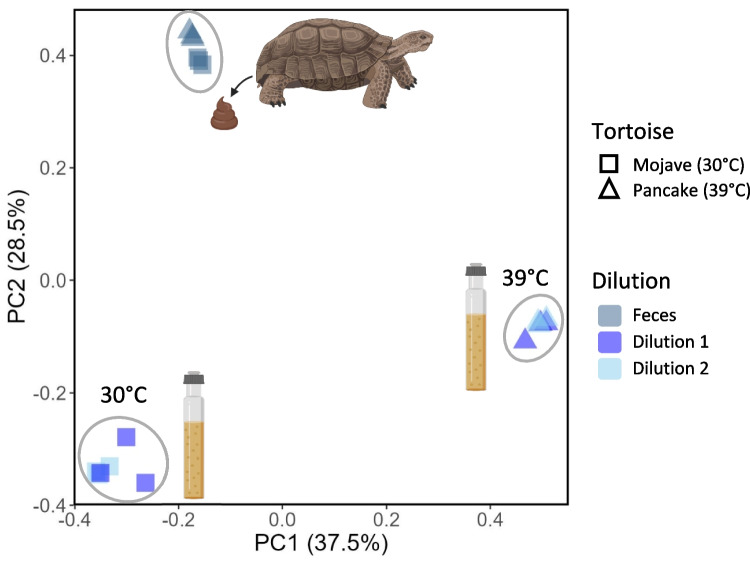
Microbial communities cluster in PCoA plots largely based on cultivation (or lack thereof) and incubation temperature (or tortoise sampled from) for the enrichments. Feces from both tortoises cluster close together. PCoA analysis was performed using the Bray–Curtis distance metric. Enrichments from feces of a male desert tortoise (Mojave) were incubated at 30 °C, and enrichments from a female desert tortoise (Pancake) were incubated at 39 °C (Fecal samples were not incubated). The circles are purely illustrative to show clustering. Images were created with BioRender.com

DNA from fecal samples was similar and clustered together in principle coordinate analysis plots, while enrichment cultures clustered together, whether the analysis used a weighted or unweighted method (Fig. 3, Supplemental Fig. 1). Feces from one tortoise were used to inoculate cultures incubated at 30 °C, and feces from another tortoise were used to inoculate communities grown at 39 °C. However, given the similarity of fecal samples from the two tortoises, the incubation temperature had a greater impact on the enrichment communities than did the differences in starting fecal samples (Fig. 3). Each of the two dilutions incubated at the same temperature was similar and clustered closely (Fig. 3).

**Fig. 4:**
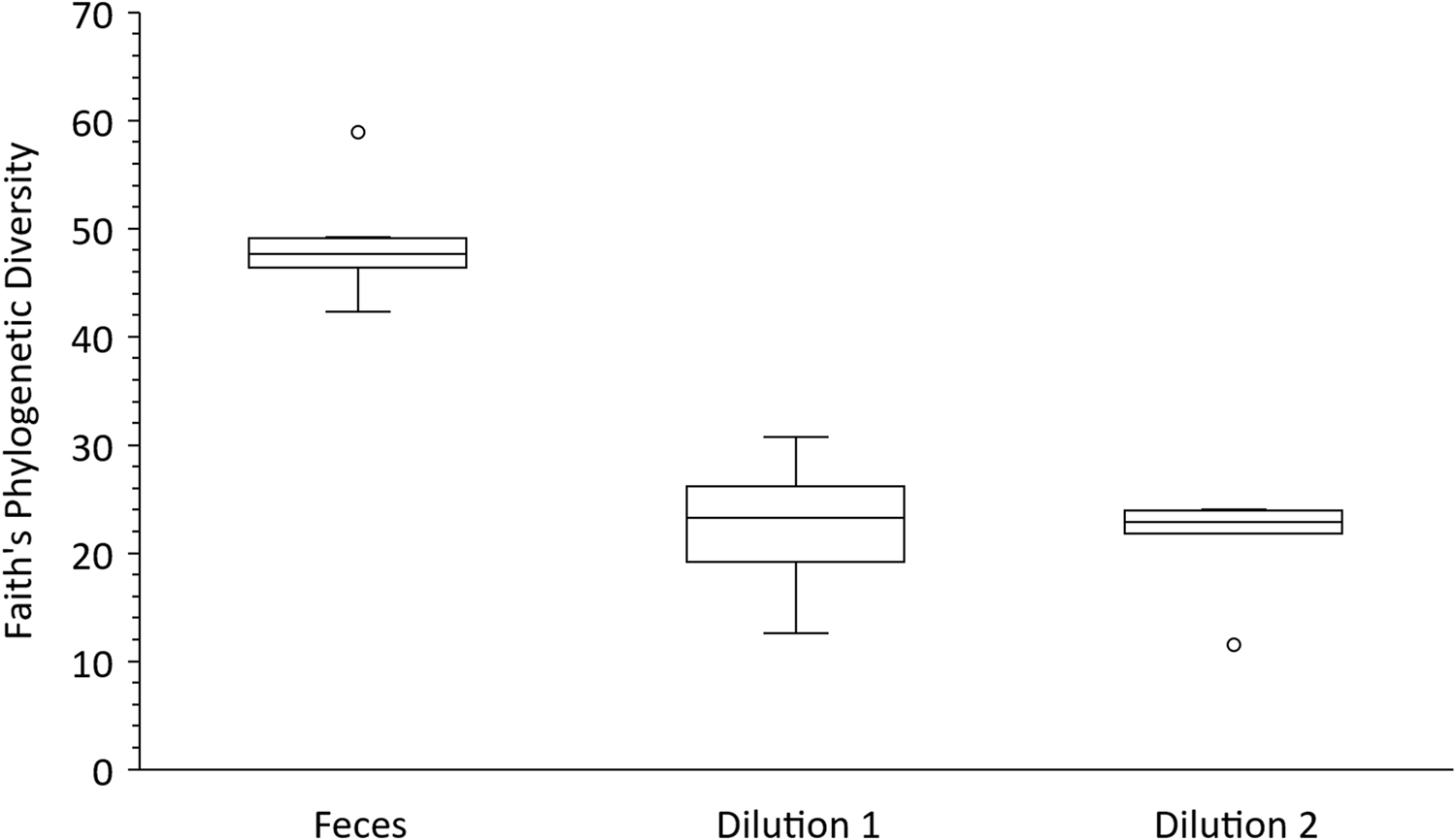
Faith’s phylogenetic diversity is significantly greater in fecal samples than in cultivated microbial enrichments. Phylogenetic diversity does not differ greatly (*p* > 0.05) between microbial enrichments, whether the starting fecal sample inoculum was less or more dilute (Dilution 1 and Dilution 2, respectively). The Kruskal–Wallis test results in a *q-*value of 0.006 between the fecal samples and each set of dilutions

Faith’s phylogenetic diversity [34] with Kruskal–Wallis tests [35] showed significantly greater diversity (*p* < 0.01) in fecal samples compared to cultivated communities, with a *q*-value of 0.006 between the fecal samples and each set of dilutions (Dilution 1 and Dilution 2) (Fig. 4). Differences in microbial communities were less pronounced with respect to the tortoise sampled from, with a *p*-value of 0.047 (Kruskal–Wallis, Supplemental Fig. 2).

### Firmicutes and Bacteroidota Dominate the Microbial Communities

Both fecal samples and cultivated communities showed large abundances of Firmicutes and Bacteroidota (Fig. 2), which are classically associated with plant biomass deconstruction [43, 44]. Enrichments incubated at 30 °C showed a greater proportion of Bacteroidota compared to their starting fecal community, many of which belong to the order Bacteroidales. Conversely, enrichments incubated at 39 °C showed more Firmicutes than their starting fecal samples (Fig. 2); this was largely due to *Enterococcus sp.,* which made up nearly 50% relative abundance or higher in microbial consortia cultivated at 39 °C (Supplemental File 1). The combined relative abundance of Firmicutes and Bacteroidota was lower for the fecal sample communities than the cultivated consortia, and several phyla present in fecal samples were less abundant or not detected in cultivated communities. Archaea were present at low abundances in all fecal samples, but they were only detected in a few cultivated samples and at lower abundances (Supplemental File 1).

Two classes within Firmicutes, Clostridia and Bacilli, are associated specifically with cellulose degradation [45, 46]. Clostridia were abundant in all samples (Supplemental Fig. 3), and Bacilli dominated in cultures incubated at 39 °C due to *Enterococcus* sp. Actinobacteria is another taxon involved in cellulose degradation [45, 46], but this class was more abundant in fecal samples than cultivated consortia (Supplemental Fig. 3).

### Cultivation Yields Enrichment of Multiple Taxa

**Fig. 5:**
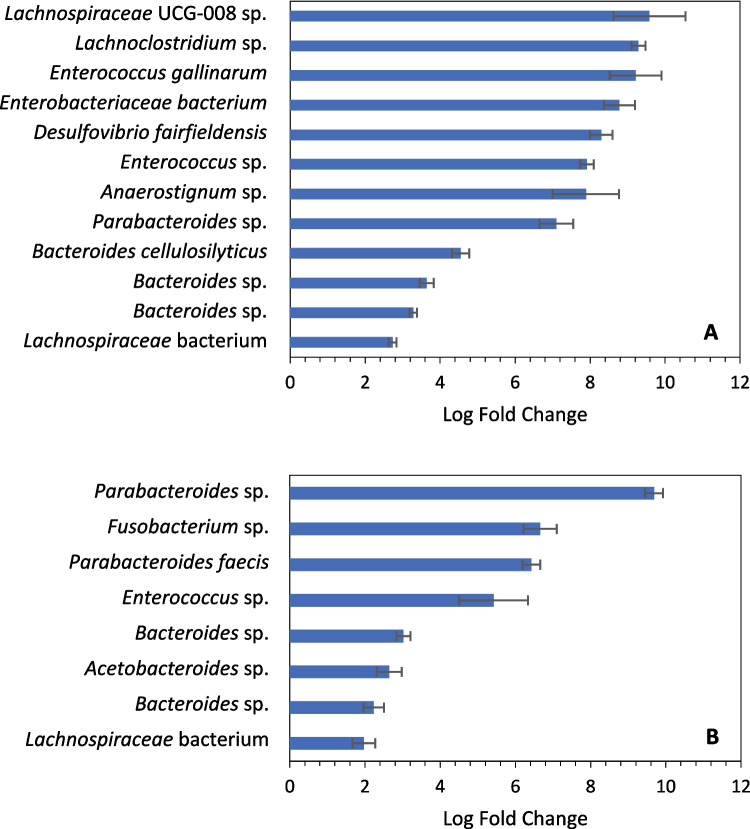
Several taxa are significantly enriched (*q* < 10^−5^) in cultivated communities compared to the fecal samples they were derived from; communities incubated at 39 °C (**A**); communities incubated at 30 °C (**B**). Results were calculated with ANCOM-BC and filtered to include only those with a *q*-value < 10.^−5^ and only taxa with an average of > 200 feature counts in the enrichments. Error bars are standard error. Note that the duplicate *Bacteroide*s sp. in each figure refer to different species. Taxa classifications and abundance counts are available in Supplemental File 1

While the overall diversity decreased for cultivated communities compared to fecal samples, certain taxa were enriched via cultivation. As mentioned before, *Enterococcus* sp. was enriched in samples cultivated at 39 °C, but it was also significantly enriched (*q* < 10^−5^ using ANCOM-BC [41]) in cultures incubated at 30 °C (Fig. 5). *Lachnospiraceae* bacteria were also significantly enriched (*q* < 10^−5^) in both cultivated communities, as were *Bacteroides* spp. and *Parabacteroides* sp. For communities incubated at 39 °C, *Enterococcus gallinarum*, *Desulfovibrio fairfieldensis*, and multiple other anaerobes were significantly enriched as well. *Bacteroides cellulosilyticus* was also more abundant in these cultivated communities, and as the name suggests, this species is associated with cellulose degradation [47]. For cultures grown at 30 °C, *Fusobacterium* sp. and *Acetobacteroides* sp. were among the especially enriched anaerobes (Fig. 5).

### Cultivated Communities Are Metabolically Active

**Fig. 6:**
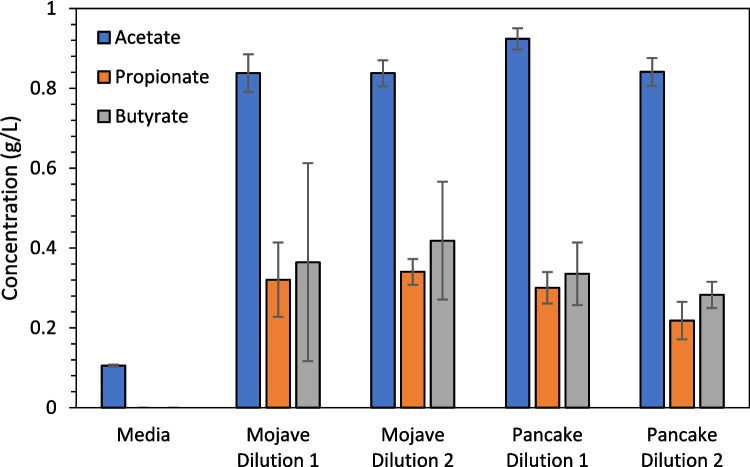
Microbial enrichments produce short-chain fatty acids. Microbial enrichments from Mojave feces were incubated at 30 °C, and enrichments from Pancake feces were incubated at 39 °C. Error bars are standard deviation of three biological replicates for enrichments and two replicates for culture media

When grown in M2 media with reed canary grass, the enrichment communities produced short-chain fatty acids, namely acetate, propionate, and butyrate (Fig. 6). Despite the different incubation conditions, the final concentrations of these products were similar. Final acetate concentrations were close to 1 g/L, while butyrate and propionate concentrations were around 0.2–0.4 g/L (Fig. 6). Firmicutes, including *Lachnospiraceae*, which were abundant in all samples, are known to make short-chain fatty acids from fibrous polymers [48], like cellulose. Thus, when grown on lignocellulose, which contains cellulose, it is expected that these communities would generate short-chain fatty acids.

## Discussion

The fecal samples used in this study came from captive animals, but in many ways, microbial membership of these desert tortoise-derived communities aligned well with fecal sample data from other tortoises, including tortoises in nature [5, 9, 42], though the foundational datasets are sparse. Firmicutes and Bacteroidota made up large portions of the microbial communities, as has been seen with gopher tortoises [9, 42] and Seychelles giant tortoises [5]. Interestingly, for Bolson tortoises, a study showed high abundances of Firmicutes but not of Bacteroidota [10]. Within Firmicutes, *Lachnospiraceae* were major members in this study and other tortoise microbial studies [5, 9, 10, 42]. High abundances of Bacteroidota and Firmicutes (including *Lachnospiraceae*) are commonly observed in other herbivore guts, like ruminants [49] and rodents [50], and these microbial members are involved in fermentation [51]. Increasing the number and diversity of tortoise microbiome datasets will help clarify other important members, characteristics, and distinguishing features of tortoise microbial communities.

In this study, alpha diversity was much higher for fecal samples compared to cultivated communities. The enrichments were more selective in membership than the fecal samples, which makes sense given challenges with cultivating different members, but also because the microbial communities were given a difficult substrate (lignocellulose) for their carbon source. While the tortoise gut is a degradative environment, it is also extremely complex [52], and many microbial members rely on other microbial fermentation processes to produce the metabolites they need to function. Thus, when culture conditions are not adequate for growth of certain microbial members or change their growth, it can affect other members within the consortium [53], and ultimately limit the overall community diversity. Additionally, cultivation impacts relative abundances of members within the community [54], and this ratio affects the metabolic profile, nutrient availability, and other conditions, like pH. Since all these conditions influence microbial growth, membership can be limited after taking an environmental sample from its native environment and cultivating the community in vitro.

Enrichment conditions appeared favorable for *Enterococcus*, given its abundance in all cultivated communities, particularly those incubated at 39 °C. *Enterococci* can be viable and resilient under many different environmental conditions [55]; while often commensal members within the gut microbiome, they can also be opportunistic bacteria [56]. Since cultivation conditions caused the community to shift from the initial fecal microbiome, it gave opportunity for *Enterococcus* sp. to dominate the consortia in many cases. *Enterococcus* spp. have also shown enzymatic activity towards cellulosic substrates [57, 58]. Other cellulose-degrading members contributed to the growth of the enrichment communities on grass, including Clostridial members. Eukaryotic lignocellulose degraders, such as anaerobic gut fungi, were also likely present, as they have been isolated from tortoise feces previously [16], but prokaryotic members were the focus of this study.

Given the abundance of *Lachnospiraceae* and their metabolic capacities [59], these members likely played a major role in converting the lignocellulosic biomass into short-chain fatty acids. Major progress recently has increased knowledge regarding this family, given its role in the gut and potential for biotechnological applications [60], but further characterization is still necessary. For example, one of the most abundant *Lachnospiraceae* members identified in this study could only be characterized to the family level (Supplemental File 1). Metagenomic sequencing is largely lacking for herbivorous reptiles; while characterized gut microbiota from other hosts are relevant and can have large portions of similar members, differences are also abundant [61]. Therefore, identifying and characterizing many members of the tortoise gut microbiome will likely require whole-genome shotgun sequencing from tortoise-associated samples.

In this study, cultivation provided evidence for microbial viability. Due to the nature of sequencing environmental DNA, it was unclear whether most of the microbial members in the fecal samples were alive at the time of sampling [62]. However, the members which grew in the cultivated communities had to be viable in the fecal samples. Since microbial communities were passaged multiple times into fresh media (10 × dilution) after the initial inoculation from fecal material, most non-viable microbes would have been washed out by the time of harvesting for DNA extraction. The production of short-chain fatty acids provided additional evidence for viability and growth of the cultivated communities. Thus, the microbial enrichments yielded insight into viable members found in the tortoise fecal communities, particularly those who grow well under mesophilic, lignocellulosic culture conditions.

## Conclusions

This study identified key members of the desert tortoise gut microbiome, with a special emphasis on the cultivable, lignocellulose-utilizing portion of the bacterial community. Bacteroidota and Firmicutes are key phyla in the community, both before and after in vitro cultivation. Alpha diversity decreased as a result of cultivation, but providing diverse cultivation conditions would likely recover taxa not supported by the initial conditions, leading to a more representative community. This study adds to the dearth of knowledge regarding anaerobes in the desert tortoise gut microbial community, but future research should utilize whole-genome shotgun sequencing to characterize novel members of this environment. Metatranscriptomic characterization would be another step to provide additional insight into microbial metabolic activity and how activities and membership change with environment. More broadly, the field would benefit from greater genomic characterization of reptilian herbivore microbial communities. The cultivation and sequencing results provided by this study illuminate key microbes within the desert tortoise gut, many of which help tortoises survive and perform their substantial roles in the ecosystem, including their cycling of lignocellulosic materials and minerals. As more data become available, it will become possible to use this and other data to make more direct links between host-associated microbes and the ecosystem as well as to apply those links to biotechnological applications.

## Supplementary Information

Below is the link to the electronic supplementary material.

Supplementary file1 (XLSX 67 KB)

Supplementary file2 (DOCX 135 KB)

## Data Availability

Raw sequencing reads have been deposited to the NIH Sequence Read Archive under the bioproject number: PRJNA1223123.
