# Supplementary material for: Microbial Enrichments Contribute to Characterization Of Desert Tortoise Gut Microbiota
Source: Microb Ecol. 2025 Jun 17;88:66. doi: 10.1007/s00248-025-02557-6 (PMC12174229; doi:10.1007/s00248-025-02557-6)
Supplement: Supplementary file 1 [file 248_2025_2557_MOESM2_ESM.docx]

**Supplemental File 1.** Abundance counts of taxa (identified at the species level where possible) found in the cultivated communities and fecal samples.

**Supplemental Table 1. Average read counts before and after filtering steps.**

|  | Before filtering | After DADA2 filtering step | After DADA2 denoising and merging | After DADA2 chimera removal | After removal of mitochondrial and chloroplast sequences and low abundance ASVs |
| --- | --- | --- | --- | --- | --- |
| Mojave Dilution 1 | 529306 | 265378 | 241629 | 24125 | 23835 |
| Mojave Dilution 2 | 537373 | 276528 | 256364 | 24532 | 24267 |
| Mojave Feces | 550816 | 254716 | 206530 | 34577 | 33498 |
| Pancake Dilution 1 | 549198 | 293585 | 279728 | 23008 | 22882 |
| Pancake Dilution 2 | 570214 | 306675 | 294095 | 25655 | 25534 |
| Pancake Feces | 566370 | 280498 | 240896 | 37775 | 36728 |

**Supplemental Table 2. Standard deviation of read counts before and after filtering steps.**

|  | Before filtering | After DADA2 filtering step | After DADA2 denoising and merging | After DADA2 chimera removal | After removal of mitochondrial and chloroplast sequences and low abundance ASVs |
| --- | --- | --- | --- | --- | --- |
| Mojave Dilution 1 | 71457 | 27749 | 23316 | 2657 | 2581 |
| Mojave Dilution 2 | 48289 | 22177 | 19826 | 2139 | 2055 |
| Mojave Feces | 21577 | 12330 | 10012 | 5189 | 5157 |
| Pancake Dilution 1 | 49699 | 28689 | 27422 | 2724 | 2717 |
| Pancake Dilution 2 | 114233 | 63903 | 61208 | 4421 | 4388 |
| Pancake Feces | 44446 | 26372 | 24746 | 4302 | 4235 |

**Supplemental Figure 1**. **Microbial communities cluster in PCoA plots largely based on cultivation (or lack thereof) and incubation temperature (or tortoise sampled from) for the enrichments**. PCoA analysis was performed using the unweighted Jaccard distance metric. Enrichments from feces of a male desert tortoise (Mojave) were incubated at 30°C, and enrichments from a female desert tortoise (Pancake) were incubated at 39°C. (Fecal samples were not incubated.)

Mojave (30°C)

Pancake (39°C)

Tortoise

Dilution

Feces

Dilution 1

Dilution 2

**Supplemental Figure 2.** **Alpha diversity is significant based on the tortoise sampled from when using a p-value cutoff of 0.05 but not a cutoff of 0.01 (p = 0.047).** The p-value was calculated using the Kruskal-Wallis test with Faith’s phylogenetic diversity.

**Supplemental Figure 3**. **Cellulose-degrading classes are abundant in fecal samples and cultivated communities.**

Mojave

Pancake

Relative Abundance (%)

|  | Bacilli | Clostridia | Actinobacteria |
| --- | --- | --- | --- |
| a | 3.47756 | 47.1067 | 12.2982 |
| b | 2.69606 | 59.4116 | 5.22833 |
| c | 2.78552 | 60.1606 | 5.96428 |
| d | 3.13126 | 35.8902 | 0.439141 |
| e | 8.33942 | 32.566 | 0.544052 |
| f | 2.07899 | 36.7199 | 0.424285 |
| g | 1.33028 | 42.9419 | 0.45755 |
| h | 0.512685 | 42.3186 | 0.481747 |
| i | 2.43866 | 38.1153 | 0.47042 |
| j | 2.0957 | 58.8138 | 1.54288 |
| k | 2.57826 | 58.0741 | 2.54345 |
| l | 2.69594 | 58.0957 | 2.11353 |
| m | 50.1529 | 26.6253 | 0.0830129 |
| n | 47.8351 | 30.6502 | 0.168626 |
| o | 54.6062 | 28.2622 | 0.175809 |
| p | 60.2802 | 25.5571 | 0.082709 |
| q | 56.9427 | 26.7374 | 0.353725 |
| r | 47.727 | 32.1818 | 0.187424 |

Feces

Dilution 1

Dilution 2

Feces

Dilution 1

Dilution 2
