# Supplementary material for: Microbial Enrichments Contribute to Characterization Of Desert Tortoise Gut Microbiota
Source: Microb Ecol. 2025 Jun 17;88(1):66. doi: 10.1007/s00248-025-02557-6 (PMC12174229; doi:10.1007/s00248-025-02557-6)
Supplement: Supplementary file 2 — Supplementary file2 (DOCX 135 KB) [file 248_2025_2557_MOESM2_ESM.docx]

**Supplemental File 1.** Abundance counts of taxa (identified at the species level where possible) found in the cultivated communities and fecal samples.

**Supplemental Table 1. Average read counts before and after filtering steps.**

|  | Before filtering | After DADA2 filtering step | After DADA2 denoising and merging | After DADA2 chimera removal | After removal of mitochondrial and chloroplast sequences and low abundance ASVs |
| --- | --- | --- | --- | --- | --- |
| Mojave Dilution 1 | 529306 | 265378 | 241629 | 24125 | 23835 |
| Mojave Dilution 2 | 537373 | 276528 | 256364 | 24532 | 24267 |
| Mojave Feces | 550816 | 254716 | 206530 | 34577 | 33498 |
| Pancake Dilution 1 | 549198 | 293585 | 279728 | 23008 | 22882 |
| Pancake Dilution 2 | 570214 | 306675 | 294095 | 25655 | 25534 |
| Pancake Feces | 566370 | 280498 | 240896 | 37775 | 36728 |

**Supplemental Table 2. Standard deviation of read counts before and after filtering steps.**

|  | Before filtering | After DADA2 filtering step | After DADA2 denoising and merging | After DADA2 chimera removal | After removal of mitochondrial and chloroplast sequences and low abundance ASVs |
| --- | --- | --- | --- | --- | --- |
| Mojave Dilution 1 | 71457 | 27749 | 23316 | 2657 | 2581 |
| Mojave Dilution 2 | 48289 | 22177 | 19826 | 2139 | 2055 |
| Mojave Feces | 21577 | 12330 | 10012 | 5189 | 5157 |
| Pancake Dilution 1 | 49699 | 28689 | 27422 | 2724 | 2717 |
| Pancake Dilution 2 | 114233 | 63903 | 61208 | 4421 | 4388 |
| Pancake Feces | 44446 | 26372 | 24746 | 4302 | 4235 |

**Supplemental Figure 1**. **Microbial communities cluster in PCoA plots largely based on cultivation (or lack thereof) and incubation temperature (or tortoise sampled from) for the enrichments**. PCoA analysis was performed using the unweighted Jaccard distance metric. Enrichments from feces of a male desert tortoise (Mojave) were incubated at 30°C, and enrichments from a female desert tortoise (Pancake) were incubated at 39°C. (Fecal samples were not incubated.)


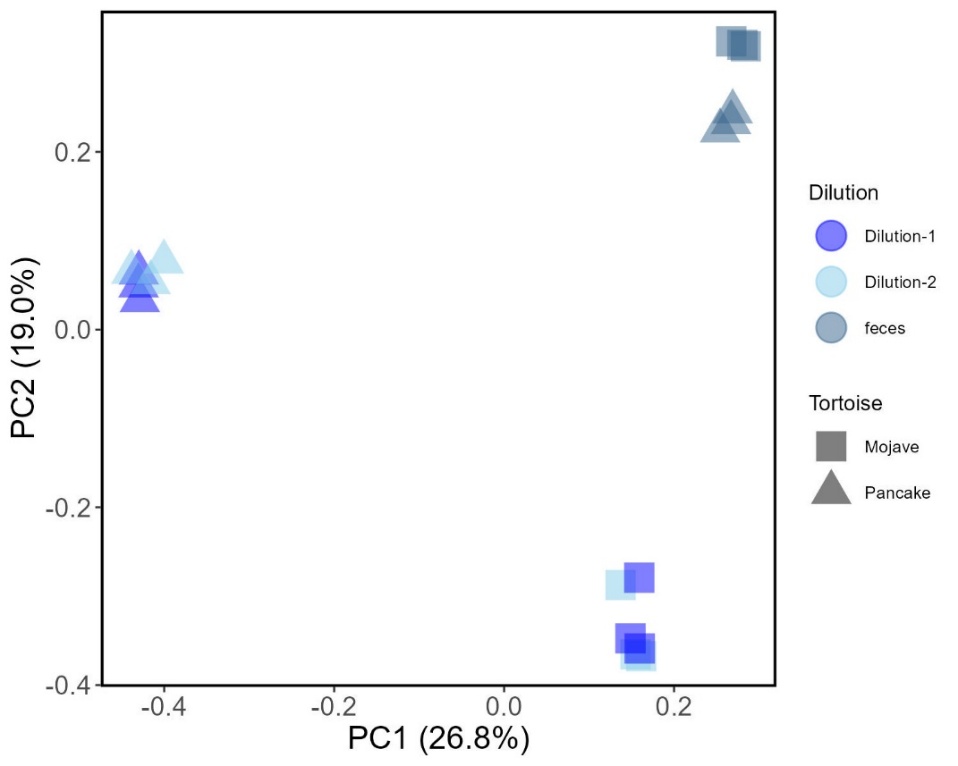


Mojave (30°C)

Pancake (39°C)

Tortoise

Dilution

Feces

Dilution 1

Dilution 2

**Supplemental Figure 2.** **Alpha diversity is significant based on the tortoise sampled from when using a p-value cutoff of 0.05 but not a cutoff of 0.01 (p = 0.047).** The p-value was calculated using the Kruskal-Wallis test with Faith’s phylogenetic diversity.


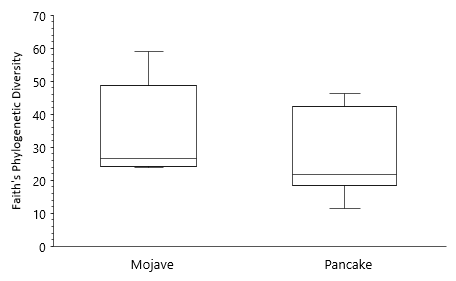


**Supplemental Figure 3**. **Cellulose-degrading classes are abundant in fecal samples and cultivated communities.**

Mojave

Pancake

Feces

Dilution 1

Dilution 2

Feces

Dilution 1

Dilution 2
